# Higher Oxygen Content Affects Rabbit Meat's Quality and Fatty Acid Profile in a Modified Atmosphere

**DOI:** 10.1155/2024/9486285

**Published:** 2024-02-13

**Authors:** Joanna Składanowska-Baryza, Dominik Kmiecik, Magdalena Rudzińska, Annalisa Vissio, Anna Grygier, Agnieszka Ludwiczak, Marek Stanisz

**Affiliations:** ^1^Department of Animal Breeding and Product Quality Assessment, Faculty of Veterinary Medicine and Animal Science, Poznań University of Life Sciences, Złotniki, Słoneczna 1, 62-002 Suchy Las, Poland; ^2^Department of Food Technology of Plant Origin, Faculty of Food Science and Nutrition, Poznan University of Life Sciences, Wojska Polskiego 31, 60-634 Poznan, Poland; ^3^Department of Agricultural, Forest and Food Science, University of Turin, Largo Paolo Braccini 2, 10095 Grugliasco, Italy

## Abstract

After 7 days of storage, the quality of the meat packed in both systems (MAP and VAC) was satisfactory; however, after 14 and 21 days, there was a noticeable decline in quality, as evidenced by changes in the water percentage and color parameters (*L*^∗^, *a*^∗^, and *b*^∗^). However, muscles stored in the MAP1 had the highest tenderness. The results indicated that as the storage time increased (up to 21 days), the share of C14:0 (*P* < 0.001) and C16:0 (*P* < 0.001) acids in the fat of the LTL muscles decreased. Only the share of C18:0 (*P* = 0.001) and C20:1 (*P* = 0.015) acids was significantly influenced by the packaging method. The highest iodine level was found in MAP1 21 days after packaging (85.68). The ratio of n-6/n-3, PUFA/SFA, AI, and TI indexes, which indicate a higher nutritional quality of fat, varied only with storage time without being affected by the gas mixture (*P* > 0.05). The H/H level differed significantly with the storage time (*P* < 0.001), with no effect of the gas mixture (*P* = 0.133). After the 21-day storage period, the controlled atmosphere led to an increase in the concentration of MUFA and PUFA and a decrease in SFA, according to the study of the fatty acid profile.

## 1. Introduction

The nutritional content and health-promoting qualities of rabbit meat have drawn the attention of researchers more and more in recent years. Since 90% of the meat is exported frozen or chilled, unfavorable microclimatic conditions along the manufacturing chain may alter the quality of rabbit meat [1]. Regarding fat and cholesterol content, rabbit meat is comparable to red meat or chicken. It is low in fat content and lean, with a high content of long-chain unsaturated fatty acids. Additionally, as meat lipids oxidize, various chemicals are formed that may have an unfavorable flavor and smell or go rancid, which is unattractive to customers [2]. In addition to apparent deterioration of taste and smell, lipid oxidation also affects the color, texture, nutritional value, and, above all, healthy safety of meat [3]. This is due to the fact that oxidation destroys valuable components, such as essential fatty acids and vitamins. The rate and direction of oxidation of meat lipids depend, among others, on their chemical composition, water content, and presence of natural pro-oxidants and antioxidants, as well as technological processes and storage conditions applied. Due to the possibility of intensive auto-oxidation of polyunsaturated fatty acids (PUFA) and the formation of unfavorable oxidation products, it is essential to ensure appropriate meat protection during long-term storage. Oxygen (O_2_) causes food spoilage due to oxidation and creates ideal conditions for developing aerobic microorganisms [4]. In our study, we analyzed the effect of two packaging methods (vacuum packaging (VAC; the most common method in small households) and modified atmosphere packaging (MAP)) and the storage period (up to 21 days) on the characteristics of rabbit meat. Furthermore, we used two gas mixtures containing different O_2_ concentrations in the study. The first is a gas mixture containing 60% CO_2_, 25% O_2_, and 15% N_2_. Manufacturers claim that this combination helps to keep the product fresh and durable for a few weeks longer. Nonetheless, most foods may absorb CO_2_ since it is readily absorbed by fat and water. MAP-packed products may exhibit collapsed packaging, flavor alterations, and a loss of juiciness due to an elevated CO_2_ content. Because nitrogen diffuses through plastic films relatively slowly and stays in the container longer, it is employed as an auxiliary or filler gas. For this reason, we selected a mixture including nitrogen and carbon dioxide. In addition, this study examined the effects of adding nitrogen to see if higher levels could be introduced in the future nitrogen to see if higher levels can be introduced in the future without affecting the organoleptic characteristics and quality of the meat. The study's second mixture comprised thirty percent carbon dioxide and seventy percent oxygen. The rationale behind utilizing this oxygen-rich combination to preserve rabbit meat for 21 days was to observe how it affected the meat's fatty acid composition and other biological characteristics. The most active element in the air that affects oxidative alterations is oxygen. Due to oxidation, oxygen (O_2_) causes food to deteriorate and fosters the growth of aerobic bacteria [4]. The choice of mixtures with 25 and 70% oxygen content and the vacuum packaging method (without oxygen) allows us to measure the beneficial effects of oxygen on rabbit meat quality. The practical outreach can be benefited by using these two distinct gas combinations. In earlier research [4], we discovered that rabbit meat quality remained acceptable after 14 days. We labeled the fatty acid profile and increased the shelf life. Moreover, while a large body of research has examined the impact of VAC or MAP on beef or chicken meat, more is needed about rabbit meat. Therefore, this study is aimed at ascertaining how the storage conditions (7, 14, and 21 days) and packaging techniques (MAP and VAC) affected the fatty acid concentration and overall quality of rabbit meat.

## 2. Materials and Methods

### 2.1. Animals

Ninety rabbits in total, crossbred between PS19 and PS59, with an average weight before slaughter of 2834 ± 81 g, were included in the study. The animals had unrestricted access to water and granulated feed with 16.0% crude protein, 14.0% crude fiber, and 10.4 MJ of metabolizable energy during the fattening phase. The animals were fasted up 24 hours before slaughter, although they were allowed unrestricted access to water. At 81 days old, the animals were slaughtered by electric stunning, which was followed by jugular vein chopping [5]. The local ethics committee's clearance was not necessary for this experiment. The hind limbs of the animals were hung to allow bleeding out.

### 2.2. Quality of Rabbit Meat

#### 2.2.1. Experimental Group

A total of 90 loins were randomly divided into ten groups: control group (CON, *n* = 9); groups stored for 7 days in VAC (VAC-7, *n* = 9), 30% CO_2_+70% O_2_ gas mixture (MAP1-7, *n* = 9), and 60% CO_2_+25% O_2_+15% N_2_ gas mixture (MAP2-7, *n* = 9); groups stored for 14 days in VAC (VAC-14, *n* = 9) and modified atmosphere (MAP1-14, *n* = 9; MAP2-14, *n* = 9); and groups stored for 21 days in VAC (VAC-21, *n* = 9) and modified atmosphere (MAP1-21, *n* = 9; MAP2-21, *n* = 9). At 24 hours postmortem, LTL from the CON group was subjected to analysis. While loins (average weight of 311 ± 15 g) of other groups were packed according to the storage method and kept at +2°C until analysis, loins were put into heat-shrinkable packaging bags PVdC/CPP made of foil polyester and polypropylene with high barrier to gases (permeability for O_2_ = 8.73 cm^3^/m^2^·24 h·0.1 MPa, 23°C, 100% RH; CO_2_ = 23.89 cm^3^/m^2^·24 h·0.1 MPa, 23°C, lt; 1% RH; and H_2_O = 4.25 g/m^2^/24 h, 38°C, 90% RH). The samples were sealed in the machine single-chamber, model PP4.2, company TEPRO S.A. (Cracow, Poland), using the vacuum range -98%. After 7, 14, and 21, LTL muscles were dissected from the loins and assessed for physicochemical characteristics and fatty acid profile. The methodology including the determination of meat acidity, color measurements, capacity to hold residual water and water fractions, and texture analysis was described in the authors' previous publication [6].

#### 2.2.2. Acidity Measurements

A combination of glass–calomel electrode (Lo 406-M6-DXK-S7/25) attached to a portable pH meter (Mettler Toledo, type 1140) and a temperature compensation probe (Mettler Toledo, model: NTC 30 k) was used to test the pH of the LTL. The pH meter was calibrated before starting each series of tests at pH 4.0 and 7.0. The following postmortem times were measured: 24 hours (pH 24 h), 7 days (pH 7 days), 14 days (pH 14 days), and 21 days (pH 21 days).

#### 2.2.3. Color Measurements

The recording was done using a portable CM 700d spectrophotometer (Konica Minolta, Amsterdam, Netherlands) with an 8 mm diameter aperture and an illuminant D65, 10° observer. Color characteristics were determined using the tristimulus CIE system, which measures lightness (*L*^∗^), redness (*a*^∗^), and yellowness (*b*^∗^) [7]. Measurements were obtained on a freshly sliced muscle surface after 45 minutes of flowering at +4°C. In the samples kept at +2°C, color measurements were repeated 24 hours, 7 days, 14 days, and 21 days after death. The device automatically determined the hue-angle and chroma values.

#### 2.2.4. Capacity to Hold Residual Water and Water Fractions

After 24 hours, 7 days, 14 days, and 21 days postmortem, the total water and free water content, cooking loss, and plasticity of LTL samples were examined.

With a few minor adjustments recommended by Pohja and Niinivaara [8], the filter paper press method, as outlined by Grau and Hamm [9], was used to assess free water (%). In summary, 0.300 g of ground beef samples was sandwiched between two glass tiles on filter paper. Each sample received a 5-minute application of 2 kg of force. Following their removal from the filter paper, the samples were promptly reweighed to ascertain any weight variations. The share of free water was calculated using the following formula: free water (%) = (sample of ground meat–sample of meat after 5 min of 2 kg pressure) 100/sample of ground meat.

The Honikel method [10] was used to calculate the percentage of cooking loss. In short, the muscles were divided into transverse slices 3 cm thick (30 g) and put inside thin polyethylene bags with the bag's wall securely attached to the sample. The meat-filled bags were submerged in a water bath set at 90°C for approximately thirty minutes or until thermocouples registered a core temperature of 75°C. After removing any remaining moisture using a paper towel, the samples were allowed to cool to room temperature. They were weighed again to find the percentage change in the sample's weight.

Meat plasticity (square centimeter) was computed using Grau and Hamm's methodology [9]. It was defined as the area of the sample of compressed beef that was used to calculate the free water content.

#### 2.2.5. Texture Analyses

The computation of the pressures and energy required to bite through the sample and cut through it was part of the texture assessment process. A Warner-Bratzler V-shaped blade was used to evaluate cutting forces and energy using a TA.XT Plus Texture Analyzer (Stable 136 MicroSystems, UK); test speed was 2 mm/s, distance was 20 mm, and force was 20 g. After computing cooking loss (kept after heat processing for 24 h), cut time at samples was calculated, and drip loss measurement was obtained to examine the cutting forces. The muscle samples were kept at room temperature before examination, and three cores with a diameter of 1.6 cm were cut out of each one using a circular knife in a direction parallel to the muscle fibers. The so-called shear energy (WB-E; N) is the area under the curve that Warner-Bratzler measured. Using Volodkevich Bite Jaws, the biting force (the force required to bite through the sample) was determined on the cooked samples (test speed 2 mm/s, strain 100%, and force 5 g). After being refrigerated in plastic bags for 24 hours at +2°C, the muscle sections used to calculate cooking loss were used to extract the meat samples for examination. The muscle segments were left at room temperature before the study. Using a Twin Blade Sample Preparation Tool, the muscles were cut into three 10 mm by 10 mm strips laterally to the muscle fibers. The force measured at the beginning of the bite (V-O; N) and the peak force (V-P; N) were computed using the slices.

#### 2.2.6. Chemical Composition

The Association of Official Agricultural Chemists' suggested approach was used to test the chemical makeup of rabbit meat [10]. The following analyses were performed 24 hours, 7 days, 14 days, and 21 days postmortem: dry matter content (the samples were dried at 105°C to constant weight); total protein content using the Kjeldahl procedure (K-424 Buchi digestion unit; Büchi Labortechnik AG, Flawil, Switzerland; Schott TitroLine, SCHOTT, Mainz, Germany); extractable fat content using diethyl ether extraction (MLL 147, ALJ Electronics, Poland); ash analyses that were conducted by AOAC protocol no. 942.05 [11]; and dry matter content that was calculated to determine the total water percent.

#### 2.2.7. Fatty Acid Methyl Ester Analysis

A previously published procedure was used for lipid extraction [12]. According to the AOCS Official procedure Ce 2-66 [13], they were first transformed into fatty acid methyl esters (FAME) to determine the amount of fatty acids. The fat samples were transesterified using sodium methylate (1 mL, 0.4 M) after being dissolved in 1 mL of hexane. The hexane layer containing FAMEs was put into a vial and subjected to GC analysis utilizing a flame-ionization detector and an SP TM-2560 capillary column (100 m, 0.25 mm, 0.2 *μ*m) (Supelco, Bellefonte, PA, USA). The column's temperature was maintained at 160 degrees Celsius for one minute. After that, it was raised to 220 degrees Celsius at intervals of 6 degrees Celsius per minute and maintained there for a further 35 minutes. Both the injection port and the detector were adjusted to 240°C. One carrier gas, hydrogen, was employed at a 1.5 mL/min flow rate. A percentage of the overall peak area of all the fatty acids was used to express the results. The retention durations of a mixture of external standard methyl esters (Supelco 37 FAME Mix C 4-C 24 Component, Sigma-Aldrich, St. Louis, MO, USA) were compared to identify the peaks. The AOCS Official technique Cd 1c-85 was used to determine the iodine value of fat.

According to Ulbricht and Southgate [14], the atherogenic (AI) and thrombogenic (TI) indexes were calculated:

(1) $$\begin{array}{c} \text{AI}=\frac{\text{C}12:0+\left(4*\text{C}14:0\right)+\text{C}16:0}{\left(\text{RPUFA}\right)+\left(\text{RMUFA}\right)},\\ \text{TI}=\frac{\text{C}14:0+\text{C}16:0+\text{C}18:0}{\left(0.5*\text{RMUFA}\right)+\left(0.5*\text{n}6\right)+\left(3*\text{n}3\right)+\left(\text{n}3/\text{n}6\right)}. \end{array}$$

The hypocholesterolemic and hypercholesterolemic ratio (HH) were calculated according to Mierlită [15]:

(2) $$\begin{array}{c} \text{HH}=\frac{\text{cis}-\text{C}18:1+\Sigma \text{PUFA}}{\text{C}12:0+\text{C}14:0+\text{C}16:0}. \end{array}$$

### 2.3. Statistical Analysis

Using the following model, the impact of the packing technique and storage duration on the meat's pH, color characteristics, water fractions, ability to retain leftover water, textural features, chemical composition (Table 1), and fatty acid profile (Table 2) was computed:

(3) $$\begin{array}{c} Y+ijk=\mu +\alpha_{i}+\beta_{j}+{\left(\alpha \beta \right)}_{ij}+{bw}_{k}+e_{ijk}, \end{array}$$

where *μ* is the overall mean of the analyzed trait, *α*_*i*_ is the fixed effect of the *i*th packaging method (*i* = 1, 2, 3), *β*_*j*_ is the fixed effect of the *j*th storage time (*j* = 1, 2, 3), (*αβ*)_*ij*_ is the interaction (packaging method *x* storage time), *b* is the partial regression coefficient, *w*_*k*_ is the loin's weight, and *e*_*ijk*_ is the random error.

The statistical software program SAS 9.4 [16] for Windows (SAS Institute Inc., Cary, NC, USA) was used to analyze the data statistically. The Tukey-Kramer correction was used when comparing least squares (LS) mean differences across multiple comparisons.

## 3. Results and Discussion

### 3.1. Value of pH

The results of pH analysis carried out in the LTL samples on 7, 14, and 21 days of storage are presented in Table 1. A previous study indicated that the tenderness and color of meat depend on the pH scale [4]. It has been shown that meat of good quality was characterized by a pH 24 value of between 5.6 and 5.9, while the pH 24 h value of medium-quality meat was 6.0–6.2 and poor-quality meat above 6.2 [17, 18]. Considering the above, the pH of meat stored in gas mixtures increased after 7, 14, and 21 days of storage in our study (*P* < 0.001). After 14 days of storage, the pH value of rabbit meat was found to be decreased (5.66–5.68), while after 21 days, there was an increase in pH (5.83–5.86) (Table 1). Despite these fluctuations, the acidity determined in the meat after 21 days was acceptable, indicating its good quality [19]. By contrast, Cullere et al. [20] found no increase in pH (*P* < 0.05) in samples during 7 and 14 days of storage in different atmospheric conditions. The results of the present study indicated that the storage period (*P* < 0.001) had a more significant influence on the pH of the meat than the analyzed packaging methods (*P* = 0.180). Contrary to the obtained results, Gariepy et al. [21] observed higher pH values in rabbit meat during storage. In their study, the acidity of rabbit meat packed in vacuum and a mixture containing 100% nitrogen was increased (6.0) after 10 days of packing and increased further with the storage period. On the other hand, meat packed in 100% CO_2_ mixture had an acceptable pH until the 25th day of storage (5.9). The authors reported that the dissolution of CO_2_ in the carcasses caused a decrease in the pH values of muscles kept in this gas. The difference in the results between the cited study and our research may be due to the use of different concentrations of CO_2_.

### 3.2. Color Coordinates

The color parameters of meat measured after slaughter indicated that it had a relatively light color (*L*^∗^ = 53.97), with the lowest proportion of the red-red (*a*^∗^ = –0.82) and a small proportion of yellow-yellow coordinate (*b*^∗^ = 5.57) (Table 1.). The lightness (*L*^∗^) parameters were found to be increased, in all studied groups after 7 (*L*^∗^_7days_ = 54.24–54.95), 14 (*L*^∗^_14days_ = 55.26–56.09), and 21 days (*L*^∗^_21days_ = 55.45‐57.20) of storage. Moreover, the general effect of storage period on *L*^∗^ was statistically significant (*P* = 0.004), but there was no significant influence on the storage method (*P* = 0.475), and there was no interaction between the packaging method and the storage period (*P* = 0.391). Vergara et al. [18] observed faster discoloration in samples stored with a higher proportion of CO_2_. On the other hand, significant (*P* ≤ 0.05) darkening was noted in muscles packed in MAP1 (*L*^∗^ = 55.45), after a 21-day storage period, compared to the samples stored in VAC and MAP2 (*L*^∗^‐VAC = 57.20 and *L*^∗^‐MAP2 = 56.68). As a result of ultrasound treatment before freezing and storing, Carrillo-Lopez et al. [22] found that lightness was significantly reduced, regardless of the exposure time (20 or 40 minutes). But after freezing and storing rabbit meat, ultrasound treatment for 40 minutes caused a significant increase in its lightness (*L*^∗^ = 56.2) (*P* = 0.010).

In the present study, the results indicated that the value of the *a*^∗^ parameter differed between the groups (*P* = 0.003). Rabbit meat packed in a mixture of oxygen and carbon dioxide was redder (*a*^∗^‐MAP1 = 0.07‐0.90) compared to the meat packed in VAC and MAP2. In addition, statistically significant differences in the value of the yellowness component (*b*^∗^) were noted between the analyzed experimental groups (VAC, MAP1, and MAP2) (*P* = 0.001). The lowest *b*^∗^ was determined in the meat packed under vacuum conditions (*b*^∗^_7days_ = 3.27, *b*^∗^_14days_ = 4.42, and *b*^∗^_21days_ = 2.73). Otherwise, the increase in the value of the *b*^∗^ parameter in MAP1 and MAP2 depended on the day of storage. On day 7, the value of *b*^∗^ was the highest in MAP2 (*b*^∗^ = 7.73), while after 14 and 21 days, it was significantly higher in MAP1 (*b*^∗^_14days_ = 8.12, *b*^∗^_21days_ = 7.33) and decreased in MAP2 (*b*^∗^_14days_ = 5.48, *b*^∗^_21days_ = 5.71).

The packaging method had also an influence on the H (*P* = 0.001), and C (*P* = 0.001) parameters, whereas time had an influence only on the *L*^∗^ parameter (*P* = 0.003) and not on others (*a*^∗^, *b*^∗^, H, and C). Moreover, there was significant interaction between the *S* × *D* in *b*^∗^ (*P* = 0.19) and C (*P* = 0.019) parameters. Vergara et al. [18] and our previous research [4] also found an inverse relationship between lightness. What is more, Vergara et al. [18] found that chroma was inversely proportional to lightness. Nevertheless, contrary to our results, they reported that both parameters significantly differed between groups only from the 15th day of storage. On the other hand, Cullere et al. [20] said that neither storage time nor packaging method influenced color coordinates.

### 3.3. Capacity to Hold Water and Water Fractions

The initial content of free water was determined at 25.03%. According to Table 1, the free water value decreased after 7 (22.88–24.40%) and 14 days (22.33–24.09%) but increased after 21 days (25.99–27.54%) of storage. It was also confirmed that the highest value of free water was achieved after 21 days (25.93-27.54%) (*P* < 0.001), with the storage method having no significant influence (*P* = 0.489). Carrillo-Lopez et al. [22] studied the effect of high-intensity ultrasound and postmortem duration on physicochemical quality. Consequently, the water holding capacity increased significantly (*P* < 0.001) as postmortem time increased (from 59.88% at 36 h postmortem to 69.61% at 156 h postmortem).

According to Table 1, the cooking loss was the highest after 7 days (28.86-29.19%) compared to 14 (24.19-26.35%) and 21 days (22.38-24.02%) in all storage treatments. By contrast, Vergara et al. [18] found that cooking loss was the highest after five days in the samples containing more CO_2_ (16.51%) compared to those containing other gas mixtures (11.10%–13.86%) and that after five days, cooking loss remained similar among the groups. 2.61 cm^2^ was the starting plasticity value, and when storage time grew, the value rose as well (*P* = 0.001). After 21 days in storage, the most plastic rabbit flesh (VAC = 3.80 cm^2^, MAP1 = 3.96 cm^2^, and MAP2 = 3.83 cm^2^) was found. Neither the duration (*P* = 0.518) nor the storage method (*P* = 0.548) impacted the overall water content. Dal Bosco et al. [23] computed a comparable figure for the meat's total water share following a 24-hour storage period at +4°C.

### 3.4. Texture

According to our results, V-O and V-P differed between the method of storage (*P* = 0.057 and *P* = 0.058, respectively) and time (*P* < 0.001) (Table 1), with no interaction between the method of storage and days (Table 1). The value of V-O and V-P was the highest in the MAP1 group, compared to the VAC and MAP2 groups. Vergara et al. [18] found the most significant changes during the first five days of storage. In the present study, we observed a decrease in V-O and V-P at 7, 14, and 21 days postmortem. Furthermore, the WB-E shear energy was influenced by time (*P* < 0.001) and decreased after 7 (4.53-5.63 N), 14 (4.69-3.74 N), and 21 days (2.76-3.68 N) in all groups of storage. Our findings agreed with those of Castellani et al. [24], who likewise observed a decline in rabbit meat that was vacuum-packed even seven days postmortem. However, the meat's shear force value tended to decline as the meat grew older, which is consistent with a study that assessed the meat's tenderness throughout storage [18, 25].

### 3.5. Chemical Composition

The initial chemical composition of rabbit meat (Table 1) was comparable to prior studies [23]. Furthermore, the amount of dry matter (*P* = 0.548 and *P* = 0.518), crude protein (*P* = 0.698 and *P* = 0.426), fat (*P* = 0.895 and *P* = 0.719), and ash (*P* = 0.887 and *P* = 0.228) in meat were all found to be significantly unaffected by the storage method or duration.

### 3.6. Fatty Acid Content

More than 20% of the total fatty acids found in rabbit meat were found to be composed primarily of three fatty acids: linoleic (C18:2), oleic (C18:1), and palmitic (C16:0) acids (Table 2). Palmitic acid (C16:0, 29.55%) was the predominant fatty acid in the LTL after 24 hours postmortem. As a member of the saturated acid (SFA) group, C16:0 acid's content is directly correlated with the carcass's fatness, which is also linked to a higher concentration of SFA and monounsaturated fatty acids (MUFA) than PUFA [26]. We discovered an *S* × *D* interaction for the fatty acid in rabbit flesh in our manuscript.

After 7 days of storage, C16:0 (31.04-33.03%) was the predominant fatty acid in the examined muscles (Table 2). Moreover, the level of C16:0 was found to be decreased at 14 and 21 days of the storage period in all storage methods (*P* < 0.001). In contrast to our findings, Dal Bosco et al. [23] showed that the share of C16:0 was comparable to the value tested in the control (24 hours after the slaughter) over 8 days of refrigerated storage. According to our study, the meat had 25.94% oleic acid (C18:1) before packing. After 7 days, the value dropped, and after 14 and 21 days, it increased (*P* < 0.001). On the other hand, no discernible variations concerning the packing technique were discovered.

According to the available literature, the average content of linolenic acid (C18:2) in rabbit meat (*M. biceps femoris*) is 777 (mg/100 g of meat) [27] and is almost nine times higher than in beef (89 mg/100 g of meat) and six times higher than lamb (125 mg/100 g of meat) and was almost three as high as the amount of this fatty acid for pork [28]. In the present study, the amount of C18:2 significantly increased at 14 (27.27-29.50%), and 21 (28.18-30.57%) days postmortem compared to other times of measurement. Furthermore, the amount of linolenic acid was the highest in the MAP1 group (30.57%), compared to VAC and MAP2 (29.79% and 28.18%, respectively).

In the VAC method, the amount of C18:3 decreased after 7 days (1.86-2.02%) but increased after 14 (2.27%) and 21 days (2.57%). In MAP1 and MAP2, the amount of C18:3 increased after 14 and decreased after 21 days. Between the packaging techniques, there were no appreciable variations in C18:3 acid (*P* = 0.649). In contrast to our findings, Chwastowska-Siwiecka et al. [1] discovered that freezing rabbit meat for up to three months resulted in an unfavorable decrease in oleic, linoleic, and *α*-linolenic acids. These variations can have resulted from the storage technique (freezing and refrigeration settings). In addition, we observed that the storage method affected C18:0 (*P* = 0.001) and C20:1 (*P* = 0.015) acids. Between 7 and 14 days, the C18:0 acid content decreased in all groups. In 21 days, VAC and MAP1 decreased (7.84% and 6.89%, respectively); however, MAP2 increased to 8.27%. In contrast, the C20:1 acid content increased in all groups and was the highest in MAP1 (Table 2). A similar result was obtained by Alasnier et al. [28], who found that during the refrigerated storage period, the amount of free fatty acids in meat increased (from 2-10 to 11-32 mg/100 g of muscle tissue). This observation mainly referred to long-chain polyunsaturated fatty acids (between 0.01 and 1.4-3.3 mg/100 g of muscle).

The LTL muscles of rabbits were characterized by a higher proportion of unsaturated acids (58.57%), including MUFA (30.59%) and PUFA (27.98%) than SFA (41.49%), compared to other species [26]. We also observed a significant interaction between *S* × *D* in SFA (*P* = 0.021) and PUFA (*P* = 0.021). The content of MUFA and PUFA decreased after 7 days of storage in all groups but increased again after 14 and 21 days.

In the present study, significant changes in the content of n-6/n-3 were found with respect to storage time (*P* = 0.009), without considering the gas mixture (*P* = 0.591). Moreover, this study found a significant difference in the PUFA/SFA ratio depending on storage time (*P* = 0.001). Also, there was an interaction between the storage method and the storage time for PUFA/SFA with *P* = 0.016.

The iodine value (CIV) significantly decreased (*P* < 0.001) after seven days of storage and was the highest in VAC (72.74), compared to MAP1 (69.19) and MAP2 (71.11) (Table 2). The CIV values in VAC, MAP1, and MAP2 increased to 77.29, 81.47, and 77.75 after 14 days. According to our research, the highest iodine level was found in MAP1 21 days after packaging (85.68). According to Leekim and Ahn [29], it is a natural phenomenon that MAP1 contains the highest CIV content because of the increased oxygen content. According to Minelli et al. [30], the higher the CIV, the higher the content of unsaturated fatty acids. Polyunsaturated fatty acids make rabbit meat susceptible to peroxidation, which results in its high iodine content.

To assess the nutritional quality of extractable fat, we analyzed the n-6/n-3 ratio, the polyunsaturated/saturated (PUFA/SFA) fatty acid ratio, and the atherogenic (AI) and thrombogenic (TI) indexes. The n-6/n-3 ratio can increase the risk of cancer and heart disease, especially blood clots that lead to heart attacks [31]. Researchers analyzing rabbit meat found that n-6/n-3 values varied significantly and ranged from 3.29 to 25 [32–34]. This result is in agreement with our results. These differences are due to varied diets, changing seasons, and the difference in the supply of n-3 acids.

According to the Department of Health and Social Security [35], the recommended ratio for PUFA/SFA should be at least 0.45, and AI and TI should be at the lowest level possible. According to our results, rabbit meat falls within the products considered healthy by the aforementioned recommendation. However, today's consumers' imbalanced fatty acid intake has been attributed to meat's PUFA/SFA ratio of around 0.1 [26]. The AI and TI ratio was significantly affected by the day of storage (*P* < 0.001), with the highest content of AI in MAP2 after seven days (0.82) and the lowest in 21 days (0.53). The TI ratio was the highest, after seven days in MAP1 and MAP2 (1.34 and 1.40, respectively), compared to vacuum (0.76). Furthermore, the TI ratio decreased in MAP1 and MAP2 after seven and increased in a vacuum after the same period.

The data on TI obtained in this study are similar to the results of Dal Bosco et al. [23], who found that the level of TI had also decreased over time (*P* < 0.05), whereas contrary to our results, Dal Bosco et al. [23] found no significant changes to AI during the 8-day storage period (*P* > 0.05). We observed an acceptable level of AI and TI in meat stored under cooling conditions for 21 days. Literature reports indicate that polyunsaturated fatty acids decrease with storage time (under freezing conditions), worsening AI and TI [36, 37]. Although fatty acid profiles of rabbit meat have been studied, little has been done to determine how storage and use affect the PUFA/SFA and AI and TI ratio.

The H/H is an index to assess the effect of fatty acid composition on cholesterol. According to Chen and Liu [38], the H/H ratio may better indicate how fatty acid composition affects cardiovascular disease than the PUFA/SFA ratio. Dabbou et al. [39] found a similar result to our result value of H/H, finding that the H/H ratio was higher in rabbit hind legs fed with Bilberry pomace additive than in rabbit hind legs fed with a commercial diet. This suggests that animal nutrition may influence hypocholesterolemic properties of their meat. Between the 7th and 21st days of storage, the samples' hypocholesterolemic/hypercholesterolemic (H/H) levels differed significantly (*P* < 0.001) without considering the mixture used. Also, the storage method and the storage time interacted (*P* = 0.050).

## 4. Conclusions

To conclude, oxygen plays the most crucial role in oxidative changes among the air components. In this study, efforts were made to show whether oxygen is an essential component of any MAP mixture used in rabbit meat packaging. It can be confirmed that meat stored for 21 days in an atmosphere with a higher concentration of carbon dioxide significantly extends the shelf life of the food (in terms of quality and fatty acid composition). However, muscles stored in the gas mixture containing 30% CO_2_ and 70% O_2_ (MAP1) had the highest tenderness. Therefore, adding oxygen to the gas mixture should be advantageous to meat tenderness. Research suggests that removing oxygen from rabbit meat mixtures and replacing it with nitrogen or argon could improve shelf life and fatty acid profile. The results of the presented study are of practical value as they are a source of knowledge about the quality of rabbit meat during its marketing.

## Figures and Tables

**Table 1:** The effect of packaging (VAC, MAP1, and MAP2), time (7, 14, and 21 days), and interaction packaging × days (*P* × *D*) on the traits of rabbit meat.

Trait	CON mean (SEM)	Packaging									RMSE	*P* values			
		VAC			MAP1			MAP2							
		7 days	14 days	21 days	7 days	14 days	21 days	7 days	14 days	21 days		*P*	*D*	*P* × *D*	Loin
pH	5.74 (0.02)	5.71^A^	5.66^Ab^	5.83^BC^	5.78^Aca^	5.68^Ab^	5.86^BC^	5.76^Aa^	5.68^Ab^	5.84^BC^	0.080	0.180	<0.001	0.878	0.711
*L* ^∗^	53.97 (0.71)	54.59^ACa^	56.09^Abb^	57.20^Bb^	54.95^AC^	55.70^AB^	55.45^AB^	54.24^A^	55.26^ABa^	56.68^BCb^	2.076	0.475	0.004	0.391	0.106
*a* ^∗^	-0.82 (0.46)	0.34^AD^	-0.76^AB^	-0.85^ACa^	0.07^ACEa^	0.90^DEa^	0.54^ADb^	-1.37^BCb^	-0.45^AEb^	-0.66^AB^	1.406	0.003	0.809	0.133	0.072
*b* ^∗^	5.57 (0.93)	3.27^AB^	4.42^AB^	2.73^A^	4.37^AB^	8.12^B^	7.33^B^	7.73^B^	5.48^AB^	5.71^AB^	2.816	0.001	0.467	0.020	0.489
H	108.1 (8.6)	110.9^AB^	109.4^AB^	123.3^Bb^	97.5^Aba^	83.9^Ab^	89.0^A^	100.4^ABa^	92.9^A^	104.7^AB^	25.02	0.003	0.341	0.841	0.308
C	5.90 (0.86)	4.00^Ba^	4.65^ABa^	3.44^Ba^	4.80^Aba^	8.22^Ab^	7.64^Ab^	7.91^A^	5.66^AB^	6.15^ABb^	2.626	0.001	0.686	0.019	0.370
Total water (%)	76.21 (0.13)	76.01	75.95	75.97	76.13	76.08	76.02	76.22	76.07	75.99	0.419	0.548	0.518	0.973	0.501
Free water (%)	25.03 (0.69)	22.88^Ab^	22.33^Ab^	27.54^B^	24.40^Aa^	24.09^Aa^	25.93^AB^	23.29^Ab^	23.26^Ab^	25.99^B^	2.047	0.489	<0.001	0.101	0.622
Cooking loss (%)	26.48 (0.55)	29.19^Ab^	25.61^ABa^	22.59^Cb^	29.74^Ab^	26.35^ABa^	24.02^Cac^	28.86^Ab^	24.19^BCa^	22.38^Cb^	1.627	0.003	<0.001	0.655	0.961
Plasticity (cm^2^)	2.61 (0.24)	3.34^AB^	3.34^AB^	3.80^B^	3.32^A^	3.34^AB^	3.96^B^	2.97^A^	2.73^A^	3.83^B^	0.693	0.116	0.001	0.763	0.542
V-O (N)	19.71 (1.66)	14.22^ACbc^	8.95^B^	7.86^B^	17.21^ACac^	11.69^BCb^	8.59^B^	15.95^ACac^	12.47^BCb^	7.77^Ba^	3.451	0.057	<0.001	0.521	0.408
V-P (N)	19.74 (1.66)	14.25^ACbc^	9.00^B^	7.89^B^	17.24^ACac^	11.74^BCb^	8.62^B^	15.98^ACac^	12.51^BCb^	7.82^Ba^	3.450	0.058	<0.001	0.527	0.441
(V-P)-(V-O) (N)	0.03 (0.02)	0.03	0.05	0.03	0.03	0.05	0.03	0.03	0.04	0.05	0.060	0.996	0.162	0.709	0.542
WB-E (N)	6.52 (0.50)	4.53^Ba^	3.74^BCb^	3.68^BCb^	5.47^Abb^	4.59^Ba^	3.30^BCab^	5.63^ABb^	4.69^Ba^	2.76^Ca^	1.210	0.325	<0.001	0.102	0.855
DM (%)	23.79 (0.13)	23.99	24.05	24.03	23.87	23.92	23.98	23.78	23.93	24.01	0.419	0.548	0.518	0.973	0.501
CP (%)	21.93 (0.22)	21.88	21.96	21.90	21.78	21.87	21.88	21.66	21.85	21.94	0.423	0.698	0.426	0.923	0.576
EF (%)	0.93 (0.04)	0.94	0.93	0.94	0.93	0.89	0.95	0.94	0.93	0.90	0.106	0.895	0.719	0.859	0.550
Ash (%)	1.10 (0.01)	1.12	1.12	1.12	1.11	1.11	1.13	1.12	1.12	1.13	0.033	0.887	0.228	0.932	0.611
W/CP	3.52 (0.03)	3.47	3.46	3.47	3.50	3.48	3.48	3.52	3.48	3.46	0.085	0.638	0.449	0.930	0.566

^A-C (a, b)^Values within a row with different superscripts differ significantly at *P* < 0.01 (*P* < 0.05). SEM: standard error of the trait; RMSE: root mean square error; Control: 0 days; VAC: vacuum; MAP1: gas mixture 1 (30% CO_2_+70% O_2_); MAP2: gas mixture 2 (60% CO_2_+25% O_2_+15% N_2_); WB-O: Warner-Bratzler force at the onset of cutting; V-O: force measured at the onset of biting; V-P: peak force; WB-E: Warner-Bratzler shear energy; DM: dry matter; CP: crude protein; EF: extractable fat; W/CP: water/crude protein ratio. *L*^∗^, *a*^∗^, and *b*^∗^ are an international designation for color parameters.

**Table 2:** The effect of packaging (VAC, MAP1, and MAP2), time (7, 14, and 21 days), and interaction packaging × days (*P* × *D*) on the fatty acid profile of rabbit meat (%).

Trait	CON mean (SEM)	Packaging									RMSE	*P* values			
		VAC			MAP1			MAP2				*P*	*D*	*P* × *D*	Loin
		7 days	14 days	21 days	7 days	14 days	21 days	7 days	14 days	21 days					
10:0	0.13 (0.05)	0.39^a^	0.20^b^	0.21^b^	0.21^b^	0.21^b^	0.21^b^	0.21^b^	0.22^ab^	0.28^a^	0.151	0.545	0.524	0.339	0.877
12:0	0.24 (0.06)	0.21^A^	0.30^AB^	0.44^Bb^	0.28^Aba^	0.24^A^	0.22^A^	0.36^AB^	0.35^AB^	0.34^AB^	0.174	0.144	0.604	0.180	0.171
14:0	2.93 (0.11)	2.76^AC^	2.72^AC^	2.29^B^	2.71^AC^	2.80^AC^	2.58^BCa^	2.76^AC^	2.68^AC^	2.24^Bb^	0.336	0.276	<0.001	0.442	0.926
14:1	0.68 (0.05)	0.65	0.69	0.67	0.66	0.74	0.70	0.66	0.60	0.70	0.166	0.592	0.796	0.687	0.142
15:0	0.18 (0.04)	0.26^AB^	0.35^Ba^	0.20^ABb^	0.24^AB^	0.22^ABb^	0.16^Ab^	0.30^ABa^	0.18^Ab^	0.39^Ba^	0.108	0.081	0.823	0.006	0.559
16:0	29.55 (0.67)	31.04 ^ACa^	28.92^AGb^	26.52^D^	31.89^AC^	29.09^AFb^	27.46^DEFG^	33.03^BCb^	27.89^DFG^	25.00^BE^	2.063	0.294	<0.001	0.049	0.980
16:1	3.25 (0.25)	1.89^b^	2.32^ab^	1.75^b^	2.00^ab^	2.49^a^	2.15^ab^	1.94^b^	2.29^b^	1.80^b^	0.630	0.365	0.015	0.949	0.826
17:0	0.69 (0.05)	0.85^ACb^	0.71^ABb^	0.76^AC^	0.88^BCa^	0.72^ABb^	0.76^A^	0.91^C^	0.71^ABb^	0.73^ABb^	0.154	0.942	0.001	0.919	0.979
17:1	0.36 (0.04)	0.35	0.36	0.36	0.36	0.34	0.34	0.41	0.26	0.38	0.107	0.969	0.256	0.337	0.129
18:0	7.76 (0.23)	9.14^B^	8.04^ACb^	7.83^A^	8.98^B^	7.61^ADa^	8.27^ACb^	8.82^BCa^	7.46^ADab^	6.89^Db^	0.655	0.001	<0.001	0.036	0.509
18:1	25.94 (0.56)	23.42^Bb^	25.16^ABa^	26.12^CE^	23.18^BC^	25.32^ABa^	26.20^CE^	23.70^Bb^	24.61^ABC^	28.36^D^	1.655	0.248	<0.001	0.074	0.154
18:2 n-6	25.07 (0.76)	26.17^ABa^	27.28^BCa^	29.79^CE^	25.31^A^	27.27^BCa^	28.18^BC^	24.18^Ab^	29.50^CDb^	30.57^D^	2.128	0.125	<0.001	0.023	0.326
18:3 n-3	2.21 (0.10)	2.02^ACa^	2.27^BCa^	2.57^Bb^	2.01^Aca^	2.35^BCb^	2.29^BC^	1.86^A^	2.59^Bb^	2.35^BCb^	0.299	0.649	<0.001	0.038	0.956
20:1	0.40 (0.07)	0.32^A^	0.32^A^	0.44^Ab^	0.24^Aa^	0.31^A^	0.43^Ab^	0.34^A^	0.33^A^	0.78^B^	0.189	0.015	<0.001	0.079	0.220
20:2 n-6	0.25 (0.04)	0.26^ab^	0.23^ab^	0.27^ab^	0.28^ab^	0.22^ab^	0.27^ab^	0.34^a^	0.21^b^	0.24^ab^	0.107	0.978	0.121	0.653	0.689
20:4 n-6	0.48 (0.11)	1.11^B^	0.60^A^	0.45^A^	1.25^B^	0.44^A^	0.74^A^	1.07^B^	0.50^A^	0.43^A^	0.329	0.387	<0.001	0.403	0.780
SFA	41.49 (0.78)	44.29^Cb^	41.08^ADa^	38.24^ADb^	44.90^BC^	40.73^ADa^	39.53^AD^	46.06^BC^	39.32^D^	35.72^E^	2.424	0.127	<0.001	0.021	0.714
MUFA	30.59 (0.65)	26.35^B^	28.73^Ab^	29.18^ACa^	26.27^Ba^	29.08^AC^	29.33^AC^	26.78^Ba^	28.02^ABb^	31.52^Cb^	1.842	0.356	<0.001	0.079	0.155
PUFA	27.98 (0.82)	29.50^A^	30.31^ADb^	32.86^BDa^	28.83^ACa^	30.20^ADb^	31.14^BCDb^	27.34^Aa^	32.74^BDa^	33.20^B^	2.289	0.217	<0.001	0.021	0.296
n-6/n-3	11.71 (0.63)	13.65^a^	12.38^ab^	11.79^b^	13.35^ab^	11.91^b^	13.05^ab^	14.16^a^	11.79^b^	13.49^ab^	1.966	0.591	0.009	0.443	0.717
PUFA/SFA	0.68 (0.03)	0.67^AE^	0.74^CE^	0.86^D^	0.65^AEa^	0.75^Cb^	0.79^BC^	0.60^A^	0.83^CDb^	0.94^Da^	0.099	0.071	<0.001	0.016	0.787
CIV	74.91 (1.28)	72.74^ABEa^	77.29^Da^	82.78^CEF^	69.19^Bb^	81.47^CDb^	85.68^Ca^	71.11^ABEb^	77.75^Da^	79.62^DF^	3.806	0.047	<0.001	0.007	0.886
AI	0.71 (0.03)	0.76^ABa^	0.68^Ab^	0.58^C^	0.78^AB^	0.69^Db^	0.63^D^	0.82^BE^	0.64^D^	0.53^C^	0.079	0.248	<0.001	0.097	0.898
TI	1.16 (0.04)	1.31^A^	1.13^C^	0.98^B^	1.34^A^	1.11^C^	1.07^BC^	1.40^A^	1.03^Ba^	0.90^Bb^	0.114	0.132	<0.001	0.014	0.905
H/H	1.66 (0.08)	1.57^CD^	1.74^CEa^	2.03^Aa^	1.50^CDb^	1.75^CBEa^	1.91^B^	1.42^D^	1.86^BE^	2.30^Ab^	0.253	0.133	<0.001	0.050	0.693

^A-E (a, b)^Values within a row with different superscripts differ significantly at *P* < 0.01 (*P* < 0.05). SEM: standard error of the trait; RMSE: root mean square error; Control: 0 days; VAC: vacuum; MAP1: gas mixture 1 (30% CO_2_+70% O_2_); MAP2: gas mixture 2 (60% CO_2_+25% O_2_+15% N_2_); CIV: iodine value; AI: atherogenic index; TI: thrombogenic index; H/H: hypocholesterolemic/hypercholesterolemic.

## Data Availability

Data used to support the findings of this study are available from the corresponding author upon request.
